# IgE Trimers Drive SPE-7 Cytokinergic Activity

**DOI:** 10.1038/s41598-017-08212-6

**Published:** 2017-08-15

**Authors:** Heather J. Bax, Holly Bowen, Rebecca L. Beavil, Raymond Chung, Malcolm Ward, Anna M. Davies, Tihomir S. Dodev, James M. McDonnell, Andrew J. Beavil, Brian J. Sutton, Hannah J. Gould

**Affiliations:** 1 Randall Division of Cell and Molecular Biophysics, King’s College London, London, United Kingdom; 2 MRC & Asthma UK Centre in Allergic Mechanisms of Asthma, London, United Kingdom; 3 NIHR Biomedical Research Centre at Guy’s and St Thomas’ NHS Foundation Trust and King’s College London, London, United Kingdom; 4 Proteomics Facility, Centre of Excellence for Mass Spectrometry, King’s College London, London, United Kingdom

## Abstract

Degranulation of mast cells and basophils, with release of agents of the allergic response, ensues when multivalent antigens bind to and cross-link the cells’ receptor-bound IgE antibodies. A widely used commercial monoclonal IgE antibody, SPE-7 IgE from Sigma, was found to possess the radically anomalous property, termed “cytokinergic”, of inducing basophil degranulation without the intervention of an antigen. We show here that the IgE monomer, freed of protein contaminants, is devoid of this activity, and that the source of the anomaly is a trace impurity, identified as a dissociation-resistant IgE trimer. Possible models for the formation of IgE trimers and the manner in which they cross-link cell surface receptors are suggested herein.

## Introduction

The function of IgE antibodies in adaptive immunity is to erect an effective barrier against infection by multi-cellular parasites and the toxicity of animal venoms^1^. These antibodies bind tightly to high-affinity receptors (FcεRI) on mast cells in surface tissues, or on basophils in the blood, to sequester the parasite antigens or toxins and evoke an immediate and potent immune response. A central feature of this mechanism is cross-linking of the receptor by attachment of a multivalent antigen to two or more receptor-bound IgEs, thereby delivering the signal for mast cell or basophil activation^2, 3^. The cost of the evolution of this protective mechanism in mammals is the risk of sensitization to otherwise harmless antigens (allergens) with the development of allergic disease. IgE binding to the receptors in the absence of antigen should not induce cell activation, which would serve no purpose in adaptive immunity, and could indeed pose a severe danger. The discovery of several monoclonal IgEs that stimulate mast cell and basophil activation without the need for an antigen was therefore perplexing^4^.

At the time of this discovery, it was already known that IgE at extremely high concentrations (5 μg/ml), some two orders of magnitude higher than that required for antigen-dependent cell activation, could up-regulate IgE receptor expression on mast cells^5–7^. Kitaura *et al*. further established that some monoclonal IgEs could induce cell activation in the absence of antigen. Different monoclonal IgEs could be classified according to their antigen-independent activities, for which they coined the term, “cytokinergic activity”^4^. The higher this cytokinergic activity of the IgE, the greater the variety and potency of the activities it evoked. Monoclonal IgEs were grouped according to whether they were highly cytokinergic (HC), moderately (MC), or poorly (PC) so. Only IgEs in the first (HC) category could induce mast cell degranulation in the absence of allergen. The most highly cytokinergic IgE was the mouse NS1 hybridoma SPE-7 IgE, an anti-dinitrophenyl (DNP) antibody^8, 9^. At 5 μg/ml the SPE-7 IgE alone activates mast cells and basophils to nearly the same extent as it does at 0.02 μg/ml in the presence of antigen, and by the same receptor cross-linking mechanism normally initiated by the antigen^4^.

In some cases, sufferers from allergic disease were found by Bennich and Johansson^10^ to have concentrations of circulating IgE of up to 5 μg/ml and it was therefore suggested that antigen-independent (“cytokinergic”) IgE action may play a part in the pathophysiology of allergic diseases^4, 11, 12^. The inhibition of cytokinergic activity by free DNP, and more recently by recombinant SPE-7 IgE Fab, pointed to the Fv domain as the seat of the cytokinergic activity of the SPE-7 IgE antibody^4, 13, 14^.

In the present study, with the aid of higher resolution from a new size-exclusion column matrix, we show that SPE-7 IgE monomers are devoid of cytokinergic activity when isolated from all contaminants. This new technology has also enabled us to demonstrate the presence of a previously unrecognized component in SPE-7 IgE preparations: an SPE-7 IgE trimer that drives the cytokinergic activity. We thus resolve a long-standing issue concerning the nature of the cytokinergic phenomenon.

## Results

**Figure 1:**
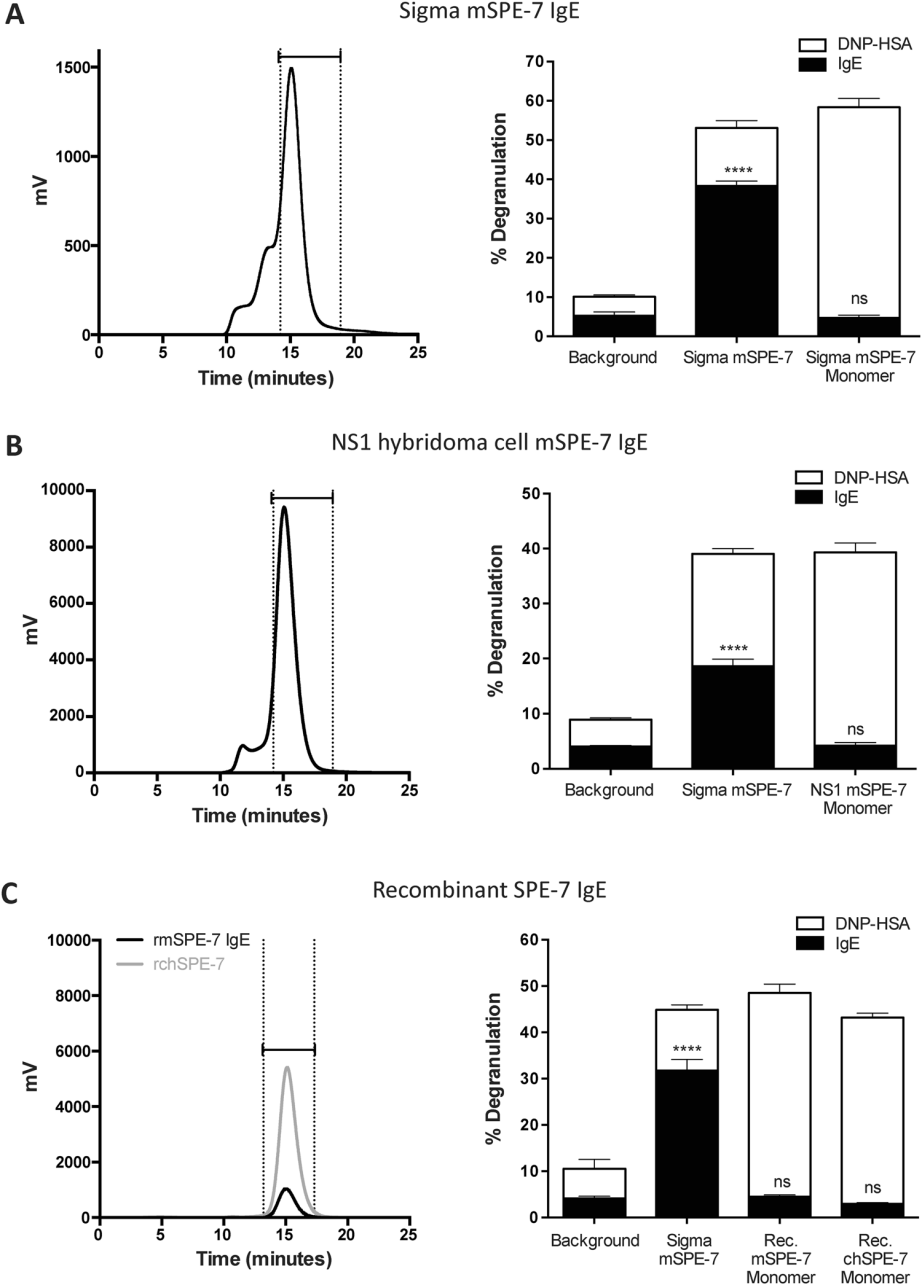
SPE-7 IgE monomer is not cytokinergic. Monomeric IgE was isolated from (**A**) Sigma mSPE-7 IgE (Sigma mSPE-7), (**B**) NS1 hybridoma cell mSPE-7 IgE (NS1 mSPE-7), and (**C**) recombinant mSPE-7 and chSPE-7 IgE (Rec. mSPE-7 and Rec. chSPE-7) preparations by size-exclusion chromatography using the Superdex S200 HPLC column. Purification profiles (left panels) show selected monomeric fractions between dotted vertical lines. Incubation with unpurified Sigma mSPE-7 IgE in the absence of antigen resulted in significant RBL-SX38 degranulation compared to buffer background control in all experiments (right panels, black lower bars; ****P < 0.0001). Monomeric IgE from all preparations did not induce degranulation of RBL-SX38 cells compared to buffer background control (right panels, black lower bars; ns P > 0.05). Functionality of the monomeric IgE antibodies was confirmed by measurement of degranulation upon cross-linking with multimeric antigen, DNP-HSA (right panels, upper white bars). Means of 3 or 4 independent experiments ± SEM are shown. Statistically significant difference to background control was determined by one-way ANOVA with Dunnett’s post-test.

To determine the origins of the exceptional cytokinergic action of SPE-7 IgE (Sigma), we isolated the pure monomeric IgE from the crude commercial product. For comparison we purified the equivalent monomeric IgE from the NS1 hybridoma (kindly provided by Dr. Zelig Eshhar) and recombinant SPE-7 produced in HEK cells using the sequence derived from the hybridoma. The recombinant SPE-7 was expressed both as wild-type mouse protein (mSPE-7) and as a mouse-human chimaeric form, comprising mouse SPE-7 IgE heavy- and light-chain variable regions and human constant regions (chSPE-7; Supplementary Figure 1). The monomers were isolated by size-exclusion chromatography on a Superdex 200 column and pooled fractions were assayed for antigen-independent activity in a rat basophil cell line expressing both the human and mouse high-affinity IgE receptors (RBL-SX38 cells). By contrast with the potent degranulating activity of the crude commercial product, none of our three purified proteins showed any detectable degranulating action (Fig. 1). At the same time, the four proteins became comparably active on addition of antigen (DNP coupled to human serum albumin, DNP-HSA). These results imply that the highly cytokinergic activity of SPE-7 may be due to a contaminating protein.

Examination of the SPE-7 product by SDS-PAGE revealed abundant contamination with other apparently unrelated proteins, amounting to 15–20% of the total protein (Supplementary Figure 2A). We were able to identify several of these components by their partial sequences obtained by LC-MS/MS, following tryptic digestion. By comparison with the sequences derived from NS1-hybridoma cells, bands 1 and 4 (Supplementary Figure 2B) were confirmed to be mSPE-7 IgE heavy- and light-chains respectively (Supplementary Figure 2C). The overall sequence coverage with the NS1-hybridoma cell-derived sequences was 68 and 89%, respectively. Contaminating bands 2, 3, 5 and 6 were also extracted and identified by LC-MS/MS to represent a range of extraneous proteins including mouse complement components C3 and C4 (Supplementary Figure 2B and Supplementary Table 1).

C3a has been reported to activate some mast cells^15–20^. Although, RBL cells are not thought to express complement receptors^21, 22^, we assayed mouse C3a in the rat basophil system to exclude degranulating activity. Degranulation was not elicited by recombinant C3a nor by the active, though not cytokinergic, recombinant IgE combined with C3a (Supplementary Figure 2D). We could therefore exclude C3a as a source of activation by the crude SPE-7 IgE.

**Figure 2:**
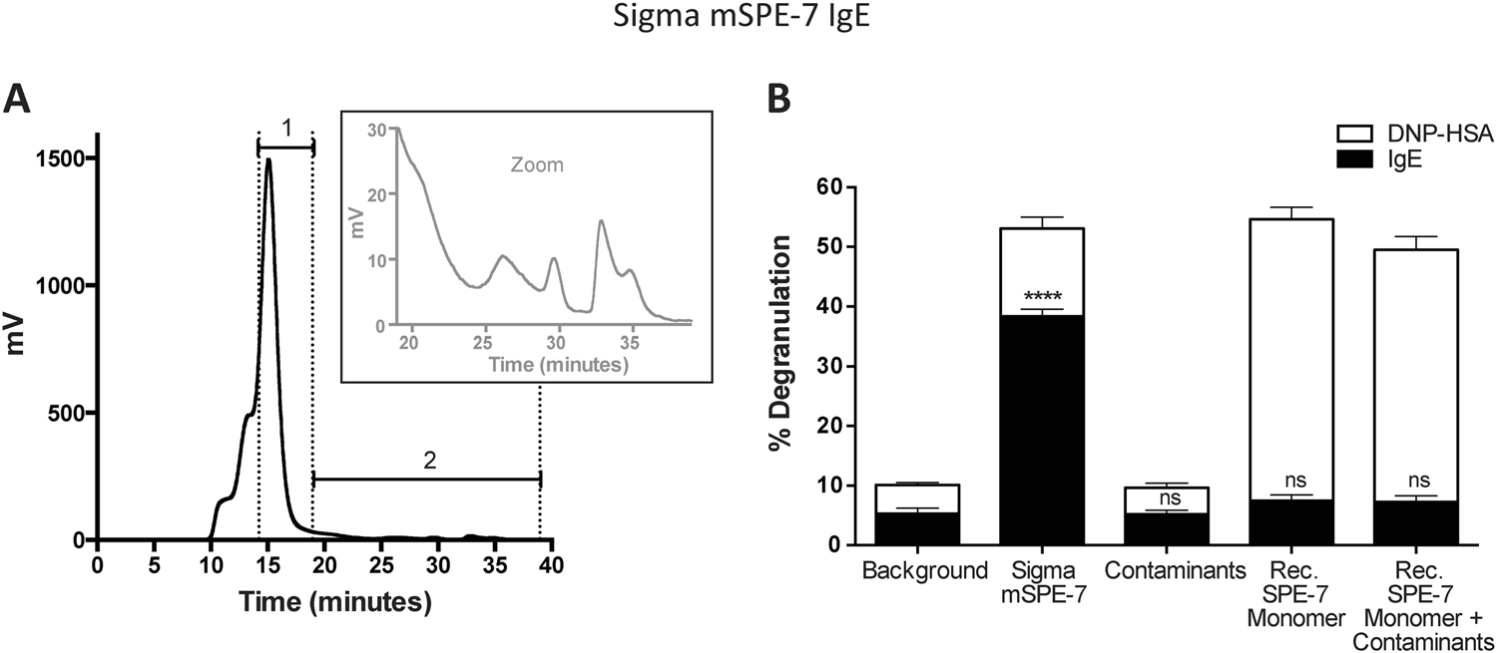
Small protein contaminants in the Sigma mSPE-7 IgE preparation are not responsible for its cytokinergic activity. (**A**) Analysis of the unpurified Sigma mSPE-7 IgE preparation by size-exclusion chromatography using the Superdex S200 HPLC column revealed the presence of small protein contaminants. Fractions smaller than monomeric IgE (fractions 1) were collected and pooled as small contaminants (fractions 2, also magnified in inset). (**B**) Although unpurified Sigma mSPE-7 IgE, in the absence of antigen, resulted in significant RBL-SX38 degranulation compared to buffer background control (black lower bar; ****P < 0.0001), the contaminants alone or in combination with 5 μg/ml recombinant SPE-7 IgE monomer, did not exhibit any cytokinergic activity (black lower bars; ns P > 0.05). Means of 3 independent experiments ± SEM are shown. Statistically significant difference to background control was determined by one-way ANOVA with Dunnett’s post-test.

Proteins smaller than IgE also appeared in the size-exclusion elution profile (Fig. 2A). These proteins, when pooled and mixed with the inactive recombinant SPE-7 IgEs, were inert in the same assay and thus also failed to account for the apparent cytokinergic activity of the crude Sigma SPE-7 IgE (Fig. 2B).

**Figure 3:**
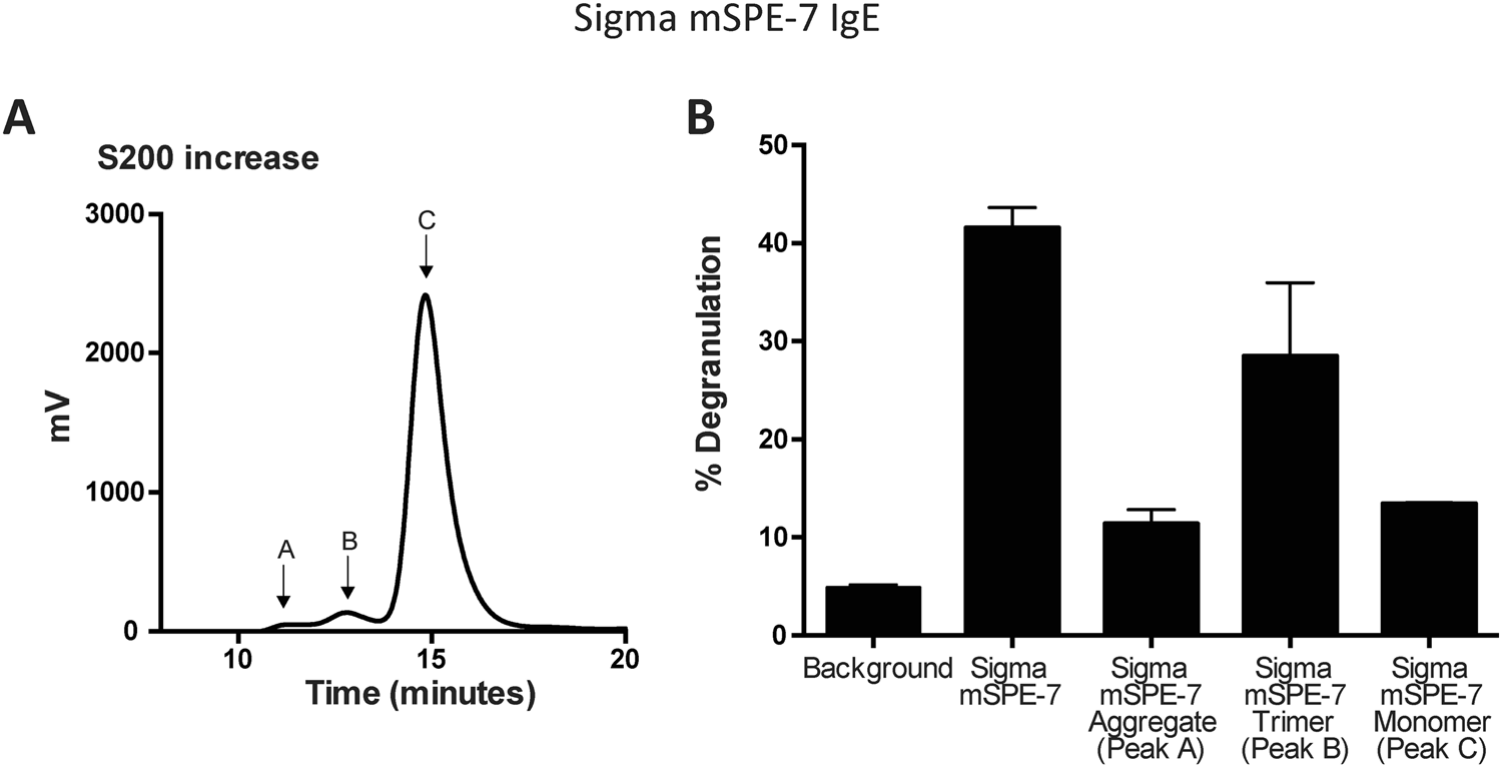
Improved size-exclusion chromatography resolves a previously unrecognized component in mSPE-7 IgE that has cytokinergic activity. (**A**) The superior resolution of SPE-7 IgE by Superdex 200 Increase compared to Superdex 200, resolved a component of intermediate size (Peak B) between the monomer (Peak **C**) and aggregated SPE-7 IgE (Peak A). (**B**) Low levels of degranulation were induced by components A and C, compared to component B. Means of triplicate technical repeats ± SEM are shown.

This left the proteins larger than the IgE monomer to be considered. The size-exclusion chromatography elution profile of IgE from the Superdex 200 column (Figs 1A and 2A) displayed, in agreement with earlier work^11, 13^, unresolved material on the leading edge of the IgE monomer zone. To achieve higher resolution in this size range we took advantage of the newly introduced, Superdex 200 Increase column matrix (Fig. 3A). On this column, Sigma SPE-7 IgE monomer was preceded by two distinct components comprising 1.6% (peak A) and 5.9% (peak B) of the total protein. Basophil degranulation assays using these components demonstrated that component B had about 65% of the activity of the unpurified Sigma SPE-7 IgE and appreciably more than that of component A or the more highly purified monomeric (component C) (Fig. 3B). At the same time, these three proteins became active on addition of multimeric antigen (DNP-HSA; data not shown).

**Figure 4:**
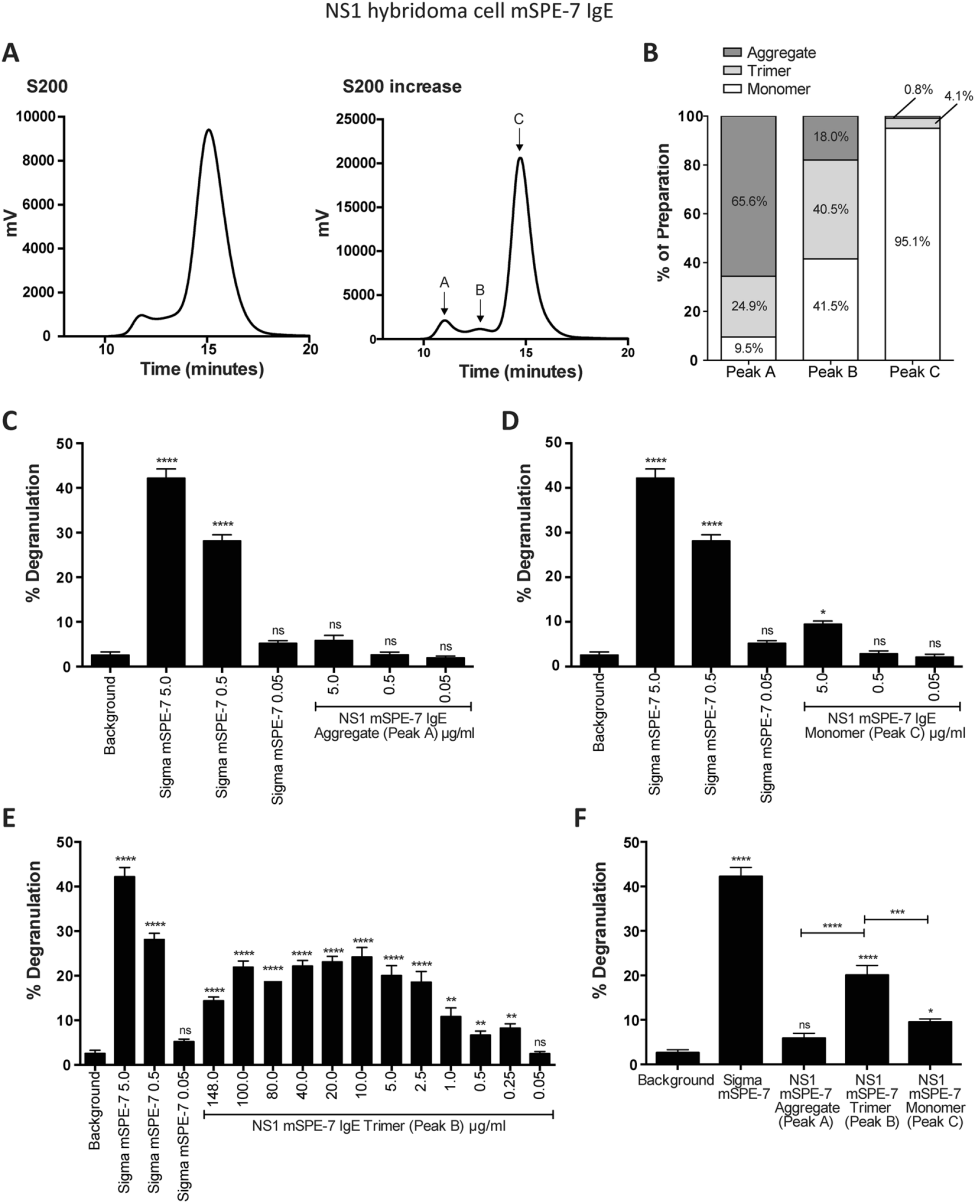
mSPE-7 IgE trimer displays cytokinergic activity. (**A**) Improved size fractionation of mSPE-7 IgE was achieved with a Superdex 200 Increase HPLC column. (**B**) SEC-MALLS analysis of peaks A, B and C from the column determined the percentage of high molecular weight aggregates, trimers and monomers in Peaks A, B and C, respectively. The percentages of each in the three fractions are indicated. (**C**) No RBL-SX38 cell degranulation above buffer background control was induced by incubation with the IgE in peak A preparation (65.6% aggregated IgE), in the absence of antigen. (**D**) Significant, but low-level degranulation, compared to buffer background control was induced by incubation with 5 μg/ml of the IgE in peak C (95.1% monomeric IgE and 4.1% trimeric IgE) in the absence of antigen. Incubation with unpurified Sigma mSPE-7 IgE, in absence of antigen, resulted in significant RBL-SX38 degranulation compared to buffer background control in both experiments. (**E**) Significant RBL-SX38 cell degranulation was induced by incubation with NS1 mSPE-7 IgE trimer in the absence of antigen (peak B in purification profile). (**F**) Degranulation induced by peaks A, B and C at 5 μg/ml is compared. Means of 3 independent experiments ± SEM are shown. Statistically significant difference to background control (unless otherwise indicated) was determined by one-way ANOVA with Dunnett’s post-test; ****P < 0.0001, **P = 0.001 to 0.01, ns P > 0.05.

Some overlap between the peaks was observed, so the preparation was scaled up using culture supernatants from the NS1 hybridoma cell line (Fig. 4). Comparison of the separations achieved by the Superdex 200 and Superdex 200 Increase demonstrates the superior resolution of the latter (Fig. 4A). Molecular mass determination by size exclusion chromatography multi-angle laser light scattering (SEC-MALLS; Fig. 3B) determined a molecular mass of 4100 ± 123 kDa for the protein in peak A, 540 ± 49 kDa for B and 180 ± 2 kDa for C. Thus component A appears to contain the IgE previously identified by others as an inactive aggregate^11, 13^. Component B contained trimers of IgE, which were partially resolved for the first time from the purified IgE monomers in peak C and the aggregate in peak A (Fig. 4B). The error in determining the molecular mass excluded the possibility of this component containing IgE dimers. SDS-PAGE analysis showed that the main components of the IgE trimer preparation, alongside the purified monomer and unpurified Sigma mSPE-7 IgE, corresponded to the IgE heavy and light chains (Supplementary Figure 3).

Peak A was, as expected on the basis of earlier work, inactive in the basophil degranulation assay (Fig. 4C). The IgE in peak C showed significantly diminished activity after purification (Fig. 4D). Nevertheless, both of these components became active on addition of multimeric antigen (DNP-HSA; data not shown). In contrast, the IgE in peak B exerted 55% of the unpurified Sigma SPE-7 IgE’s activity, and this activity was significantly greater than that of the IgE in peaks A and C (Fig. 4E and F). We conclude that the cytokinergic activity requires a previously unrecognised component of IgE trimer in the unfractionated Sigma SPE-7.

The above results leave no doubt that monomeric SPE-7 IgE has of itself no cytokinergic activity, or any doubt about the source of this activity in the Sigma IgE product, and most probably therefore in other monoclonal IgE preparations displaying cytokinergic activity at lower levels. The results nevertheless raise further questions, as discussed below.

## Discussion

Reports that basophils or mast cells respond to high concentrations of certain monoclonal IgE antibodies date back to 1985^5^. Sensitization with these IgE antibodies manifests itself as a rise in cell signalling, leading to up-regulation of the high-affinity IgE FcεRI surface receptors on mast cells and basophils, increased cell survival, and cytokine secretion. These all occur in varying degrees, depending on the antibody^4, 9, 11^. Kitaura *et al*. coined the term “cytokinergic” for this erratic phenomenon, and classified the responsible IgEs according to the magnitude of the response as high (HC), moderate (MC) or poor (PC)^4^.

A single antibody preparation, the commercial mouse hybridoma SPE-7 anti-DNP IgE from Sigma, stands at the high-response extreme of the HC group, and causes not only pre-sensitization to antigen activation, but also degranulation of basophils and mast cells in the absence of antigen^4, 13^. This was not observed in the earlier study with this antibody^11^, but was confirmed in subsequent published work^14, 23–28^, and also here. The reason for this discrepancy has not been resolved.

This highly cytokinergic activity was confined to a small sub-group of the monoclonal IgEs, but it appears to violate a tenet of cellular immunology, that cell activation is initiated by cross-linking and clustering of, in this case, IgE-bearing surface receptors on binding of multivalent antigens. This raises the question of how such cross-linking might occur without intervention of a divalent or multivalent antigen.

One plausible explanation was implicit in the results of Kalesnikoff *et al*. and Kitaura *et al*.^4, 11^, namely, the existence of a secondary (weak) IgE binding site in the Fab arms of the receptor-bound SPE-7 IgE. This would capture a free, unbound IgE from the supernatant that could attach itself to receptor-bound IgEs so as to form a cross-link, thereby simulating the action of a divalent antigen. In support of this scheme is the rapid termination of the HC activity by (1) removal of free SPE-7 IgE from the medium^13, 29^, or (2) addition of monovalent DNP hapten or recombinant SPE-7 IgE Fab^13, 14^. Furthermore, we have shown that the postulated cross-linking occurs through an Fv/Fv interaction^14^. These features are strongly reminiscent of an idiotype-anti-idiotype (Id-anti-Id) reaction. Intriguingly, SPE-7 IgE has been shown to display multi-specificity, binding not only to DNP, but also to a totally unrelated protein antigen^30, 31^. The ‘conformationally labile’ CDRs may be responsible for the occurrence of this Fv/Fv (protein-protein) interaction.

A mechanism by which the exceptionally high expression of the FcεRI-bound monoclonal IgE might enhance even a low affinity interaction between the free and bound IgE was proposed^32^. The authors envisaged an un-physiological situation in which a self-reactive monoclonal IgE, saturating the receptor binding sites on the cell surface, are exposed to a large excess of the same IgE. Self-association between the free and bound IgEs would be strongly enhanced by the large number (5 × 10^5^) of the receptors and the mobility of the IgE-receptor complexes on the cell surface.

Attempts in previous studies to free the SPE-7 IgE monomer of all traces of oligomers, which upon binding to the surface receptors could cause FcεRI clustering, were evidently insufficient to eliminate the trimers that we have now separated by affinity chromatography followed by a superior size-exclusion chromatography matrix (Fig. 4A).

The primary outcome of the results presented here is that the IgE monomer in the widely used SPE-7 Sigma product has no detectable cytokinergic activity. It would not be surprising if the same conclusion were, by extension, equally true for other monoclonal IgE preparations falling into the HC class. Our results further suggest that the outcome of earlier work on supposedly cytokinergic IgE preparations may have been vitiated by inadequate resolving power of the methods available at the time and may need to be re-evaluated.

The conclusion that a trimer is responsible for the degranulating action of the SPE-7 IgE preparation raised a number of new questions. We may ask in the first place why the trimer is the only IgE oligomer that is formed. A linear self-association process would lead to dimers, trimers and higher forms in predictable proportions, depending on the subunit concentration, as seen for example in negative-stain electron microscopy (EM) of antigen-IgE complexes^33^. However, in this recent study, as also in earlier EM investigations of self-associating (Id-anti-Id) or cross-linked (by divalent haptens) IgG^34–37^ or IgE molecules^38^, a preponderance of cyclic dimers, trimers or tetramers, rather than linear complexes, was observed. A feature of the IgG structure, missing in IgE, is the presence of a flexible “hinge” between the Fab and Fc regions; this allows the angle between the Fab arms to vary widely, permitting formation of a range of cyclic structures. However, the more restricted flexibility of IgE, lacking a hinge region, might be expected to limit the possible oligomers that can form. Cyclic oligomers are thermodynamically favoured provided that the structures are not strained in ring formation.

A further key factor is the location of the epitope recognised by the self-associating antibody; in the study by Gieras *et al*.^33^, the relative disposition of epitopes presented on a model antigen dictated the types of complexes formed, including ring dimers or tetramers of IgE depending on the antigen. In the earlier Id-anti-Id study^38^, ring dimers and tetramers were again formed, although these consisted, respectively, of 1:1 and 2:2 complexes of IgE (Id-bearing) and IgG1 (anti-Id) antibodies. These studies indicate that IgE molecules alone can accommodate the formation of cyclic dimers or tetramers, and thus presumably also trimers, depending on the location and orientation of the epitope.

We have previously modelled the IgE Fab arms relative to the known structures of both free and receptor-bound IgE-Fc^39, 40^ and found that they are more restricted in their conformations than the more flexible IgG Fab arms. Furthermore, the range of inter-Fab angles in the models agrees well with the measured EM images: 141° ± 34° ^38^. Thus we can infer that SPE-7 IgE trimer formation involves each antigen-binding site recognising a determinant that we have shown is located within the Fv region^14^; the precise location of the epitope and the restricted flexibility of the SPE-7 IgE Fab arms may confine self-association to trimers alone. The thermodynamic stability of a cyclic structure, as observed in the earlier EM studies, is apparently sufficient to survive dilution during chromatography.

While we have shown that the antigen-independent degranulating activity of SPE-7 resides in the trimer fraction, we cannot distinguish whether the trimers act directly upon the mast cells or interact with receptor-bound SPE-7 IgE monomers. This cannot be addressed experimentally since we have so far been unable to eliminate residual monomers from our trimer preparation. In any case, without knowing the precise structure of the trimer, it is impossible to predict whether the three SPE-7 IgE-Fc regions could engage three receptors simultaneously and lead to cell activation. In the unfractionated commercial SPE-7 samples, monomer predominates and must saturate the receptors, so that for the trimers to engage with receptor-bound monomer, we must assume that a cyclic trimer is capable of dissociation into an open structure.

We are confronted next with the problem of the circumstances under which the trimers are formed. Local concentration must be assumed to play a part. The SPE-7 IgE is a mouse hybridoma, and is synthesized in mouse ascites cells or in tissue culture, where the concentrations may favour self-association of the IgE. There may be other features of the manipulations that the preparations undergo during synthesis, purification, concentration, freezing and storage.

A final question is whether IgE trimers exist *in vivo*. Advocates of cytokinergic IgE have pointed out that the concentrations required for SPE-7 to provoke basophil degranulation may be reached in individuals infected by certain parasites or suffering from allergic disease. However, as previously pointed out^32^, HC IgE activity was discovered in experiments with monoclonal IgEs. Situations in which IgE antibodies of a single specificity are generated at high concentrations must be quite rare, perhaps confined to monoclonal responses in germinal centre-like structures in tissues. Nevertheless, it has been shown that certain monoclonal IgE antibodies, notably SPE-7, can elicit antigen-independent mast cell activation in the skin of mice during sensitization to chemical haptens^41^.

The cell degranulating action of SPE-7 IgE observed here conforms to the original definition of cytokinergic IgE insofar as it is antigen (DNP)-independent, yet it seems to involve self-association in the manner of a trimeric Id-anti-Id complex, rather than being an activity of monomeric IgE molecules.

## Methods

### SPE-7 cDNA synthesis and sequencing

RNA was extracted from SPE-7 IgE hybridoma cells (NS1 cells; kindly provided by Prof. Zelig Eshhar^8^), and the immunoglobulin heavy and light chains for SPE-7 IgE were amplified from hybridoma cDNA by PCR as previously described^14^. The variable region sequences are deposited in GenBank (Accession nos. KJ734989 and KJ734990).

### Recombinant SPE-7 IgE PIPE cloning and expression

Polymerase incomplete primer extension (PIPE) cloning^42^ was used as previously described^14, 43^ to clone murine and chimeric SPE-7 IgE antibodies into pVITRO1 vector plasmids (Invivogen).

Human embryonic kidney cells (HEK293-F suspension FreeStyle™ cells; Invitrogen) were transfected with purified plasmid DNA using PEI (Sigma-Aldrich) and cultured in Dulbecco’s Modified Eagle medium (DMEM), supplemented with 10% FCS, 2 mmol/L L-glutamine, 10 U penicillin/streptomycin (all Invitrogen) at 37 °C and 5% CO_2_. Following selection with 50 ng/ml hygromycin B (Invitrogen), cells were cultured in FreeStyle™ 293 expression media (Invitrogen), supplemented with hygromycin B, in spinner culture flasks. Culture supernatants were harvested and filtered through a 0.45 μm cellulose acetate filter.

### ELISA for detection of recombinant murine and human SPE-7 IgE expression and DNP specificity

Recombinant murine and chimaeric SPE-7 IgE (rmSPE-7 and rchSPE-7) protein expression and DNP-specificity was confirmed using ELISA plates (Maxisorp; Nunc) coated overnight with 5 mg/ml DNP-HSA (Sigma-Aldrich), blocked with PBS-1% BSA, and incubated with cell-free culture supernatants for 2 hours. This was followed by addition of HRP-conjugated anti-human IgE detection antibody diluted 1/5000 (Sigma), or anti-mouse IgE detection antibody diluted 1/2000, and then avidin-HRP diluted 1/1000 (both R&D Systems). IgE was detected with TMB (R&D Systems) by measuring absorbance at 450 nm.

### SPE-7 IgE from the NS1 hybridoma cell lines

SPE-7 IgE hybridoma cells (NS1 cells; kindly provided by Prof. Zelig Eshhar^8^, were cultured in spinner culture flasks in Dulbecco’s Modified Eagle medium (DMEM), supplemented with 15% heat inactivated horse serum, 2 mmol/L L-glutamine, 10 U penicillin/streptomycin (all Invitrogen). Culture supernatants were harvested and filtered through a 0.45 μm cellulose acetate filter.

### Purification of the monomeric and oligomeric SPE-7 IgE antibody and contaminating proteins

The recombinant mSPE-7 and chSPE-7 IgE antibodies, and mSPE-7 IgE from the NS1 hybridoma culture were isolated from cell culture supernatants using an HiTrap NHS-activated HP column (GE Healthcare) amine coupled with IgG4-Fc(sFcεRIα)_2_ fusion protein^44^ and eluted with 0.2 M glycine, pH 2.5, before immediate neutralization with 1 M Tris, pH 8.6. Monomeric IgE antibodies were isolated by size-exclusion chromatography using a Superdex™ 200 column (Amersham Pharmacia Biotech) on an HPLC system (Gilson). For the designated experiments, the same purification methods (affinity chromatography and size exclusion) were used to purify the commercially available Sigma SPE-7 IgE and smaller contaminating proteins. Monomeric IgE antibodies were eluted at around 15 minutes and the contaminants were eluted after 20 minutes.

A new technology, the Superdex™ 200 Increase column (Amersham Pharmacia Biotech) was also utilized on the HPLC system (Gilson) to fractionate SPE-7 IgE produced from the NS1 hybridoma cell culture, which had first been isolated by means of the Fc(sFcεRIα)_2_ fusion protein, and the commercially available Sigma SPE-7 IgE.

### SDS-PAGE and liquid chromatography tandem mass spectrometry (LC-MS/MS)

SDS-PAGE electrophoresis was performed in 3-(*N*-morpholino) propane sulfonic acid (MOPS) buffer (Novex, Life Technologies) at 180 V for approximately 90 minutes. The gel was fixed and stained with Brilliant Blue G Colloidal stain (Sigma), and then de-stained to reduce background staining before a scanned image was recorded.

In-gel reduction, alkylation of cysteines and proteolytic digestion with trypsin (Promega) were performed on excised gel sections prior to liquid chromatography tandem mass spectrometry (LC-MS/MS) analysis using a Thermo Scientific Orbitrap Velos Pro™ mass spectrometer coupled to a Proxeon EASY-nLC II nano-flow liquid chromatography system. Eluting peptides were ionised by electrospray ionisation. Precursor ions were surveyed between m/z 350–1800 at a resolution of 30000 and the twenty most intense ions were subjected to MS/MS by collision-induced fragmentation in the iontrap with a normalised collision energy value of 35.

Raw data from the mass spectrometry analysis were processed using Proteome Discoverer™ software, version 1.4 with Mascot version 2.2 as the search algorithm. Initially, the data from gel bands 1–6 were searched against the uniprot_sprot_130220 database specifying mouse proteins (16597 entries). Then the data from selected gel bands, namely 1 and 4 only, was searched against an in-house custom database (128 entries), comprising sequences for SPE-7 IgE heavy chains and light chains derived from NS1-hybridoma cells. Finally, all database search results were reviewed within Scaffold 4.3.2 (Proteome Software) where protein identifications resulting from both the mouse protein database search and matches against the specific heavy and light chain SPE-7 IgE sequences, were filtered using a 95% confidence score and a minimum of three matched peptides to generate the overall protein sequence coverage.

### SEC-MALLS

The three peaks isolated from the S200 Increase HPLC purification of NS1 mSPE-7 IgE were run again on the Superdex™ 200 Increase column (Amersham Pharmacia Biotech) to determine their molecular weight, using an inline miniDAWN light-scattering detector and an Optilab DSP Interferometric Refractometer, and the data was analysed using the ASTRA 4.9 software (Wyatt Technology).

### Mast cell activation assays

RBL-SX38 cells, which express both the native rat FcεRI and the human tetrameric form of FcεRI (kindly provided by Prof. Jean-Pierre Kinet^45^), were cultured in RPMI supplemented with 10% FCS, 2 mmol/L L-glutamine, 10 U of penicillin/streptomycin, and 50 μg/mL Geneticin (all Invitrogen) at 37 °C, 5% CO_2_. Cells were seeded at 1 × 10^4^ cells/100 μl/well in a 96-well plate in complete media omitting Geneticin and cultured at 37 °C, 5% CO_2_ overnight before stimulation.

Following washing with HBSS/1% BSA, adherent cells were stimulated with IgE antibodies (diluted in HBSS/1% BSA to 5 μg/ml in 100 μl/well, unless otherwise stated) for 4 hours at 37 °C, 5% CO_2_. Sigma SPE-7 IgE was used directly from the vial (Sigma mSPE-7) or following HPLC chromatography. Cross-linking of IgE-bound FcεRI was carried out by incubation with 0.1 μg/ml DNP-HSA diluted in HBSS/1% BSA (Sigma-Aldrich) for 1 hour at 37 °C, 5% CO_2_. Alternatively, mast cells were stimulated with up to 5 μg/ml recombinant murine C3a (Bio-Techne) alone or in combination with recombinant SPE-7 IgE.

After each incubation, supernatants were harvested for analysis. Cells were finally lysed with 100 μl/well HBSS/0.5% Triton X-100 (Sigma-Aldrich) for 30 minutes at 37 °C, 5% CO_2_ and the lysates collected. β-hexosaminidase release was measured using 4-methylumbelliferyl N-acetyl-β-D-glucosaminide substrate (Sigma-Aldrich) as previously described^46^ and degranulation expressed as a percentage of the total for each well. Statistically significant difference between experimental conditions was determined by one-way ANOVA with Dunnett’s or Tukey’s post-test. GraphPad Prism (GraphPad Software, Inc.) was used for all statistical analyses.

### Data Availability Statement

All data generated or analysed during this study are included in this published article (and its Supplementary Information files).

## Electronic supplementary material

Supplementary File
