# Supplementary material for: IgE Trimers Drive SPE-7 Cytokinergic Activity
Source: Sci Rep. 2017 Aug 15;7:8164. doi: 10.1038/s41598-017-08212-6 (PMC5557881; doi:10.1038/s41598-017-08212-6)
Supplement: Supplementary file 1 — Supplementary File [file 41598_2017_8212_MOESM1_ESM.pdf]

SUPPLEMENTARY INFORMATION

IgE Trimers Drive SPE-7 Cytokinergic Activity

Heather J Bax[1,2], Holly Bowen[1,2,3], Rebecca L Beavil[1,2], Raymond Chung[4], Malcolm Ward[4], Anna M Davies[1,2], Tihomir S Dodev[1,3], James M McDonnell[1,2], Andrew J Beavil[1,2], Brian J Sutton[1,2], Hannah J Gould[1,2,3]

Supplementary Figure 1 Production of recombinant SPE-7 IgE antibodies. (A) Schematic of murine and human chimeric SPE-7 IgE antibodies, rmSPE-7 and rchSPE-7 respectively, with murine SPE-7 VH and VL domains (white) and either murine (black) or human (grey) constant domains. (B) DNP-specificity of both recombinant IgE antibodies was confirmed by ELISAs with DNP-BSA used to capture the antibodies in cell culture supernatants and binding detected by using anti-murine IgE and anti-human IgE antibodies, respectively. (C) Purified recombinant SPE-7 antibodies were analysed by SDS-PAGE alongside the unpurified Sigma mSPE-7 IgE preparation.

Supplementary Figure 2 Contamination of Sigma mSPE-7 IgE. (A) The unpurified Sigma mSPE-7 IgE preparation was titrated on an SDS-PAGE gel and densitometry used to determine the percentage of the contamination band (indicated by *) of the total protein. An average 17% contamination was calculated. (B) Following SDS-PAGE analysis of Sigma mSPE-7 IgE under denaturing protein bands 1-6 were excised for evaluation by LC-MS/MS. (C) The overall sequence coverage with NS1 hybridoma cell-derived sequences is indicated (amino acids shaded grey indicates where coverage was achieved). (D) The activity of the Sigma mSPE-7 IgE preparation contaminant, murine C3a, was evaluated in an RBL-SX38 mast cell degranulation experiment. Murine C3a alone (left panel; black lower bars) or in combination with recombinant SPE-7 IgE (rSPE-7, shown to be non-cytokinergic in Fig. 1C; right panel; black lower bars) did not induce any mast cell degranulation above buffer background control, whereas significant degranulation was induced by the unpurified Sigma mSPE-7 IgE preparation (Sigma mSPE-7; both panels; black lower bars). Means of 3 independent experiments ±SEM are shown. Statistically significant difference to background control was determined by one-way ANOVA with Dunnett's post-test; **** $P<0.0001$; ns $P>0.05$.

Supplementary Figure 3 Analysis of SPE-7 IgE preparations. NS1 hybridoma cell mSPE-7 IgE peak B (containing trimers) and re-purified monomer peak were analysed by SDS-PAGE alongside the unpurified Sigma mSPE-7 IgE preparation. Protein bands corresponding to IgE heavy and light chains were identified in all preparations.

Supplementary Table 1 Summary of protein identifications by LC-MS/MS. A list of proteins identified at over 95% confidence score within Sigma mSPE-7 IgE gel bands 2, 3, 5 and 6 (gel shown in Supplementary Fig. 2B).

Supplementary Figure 1

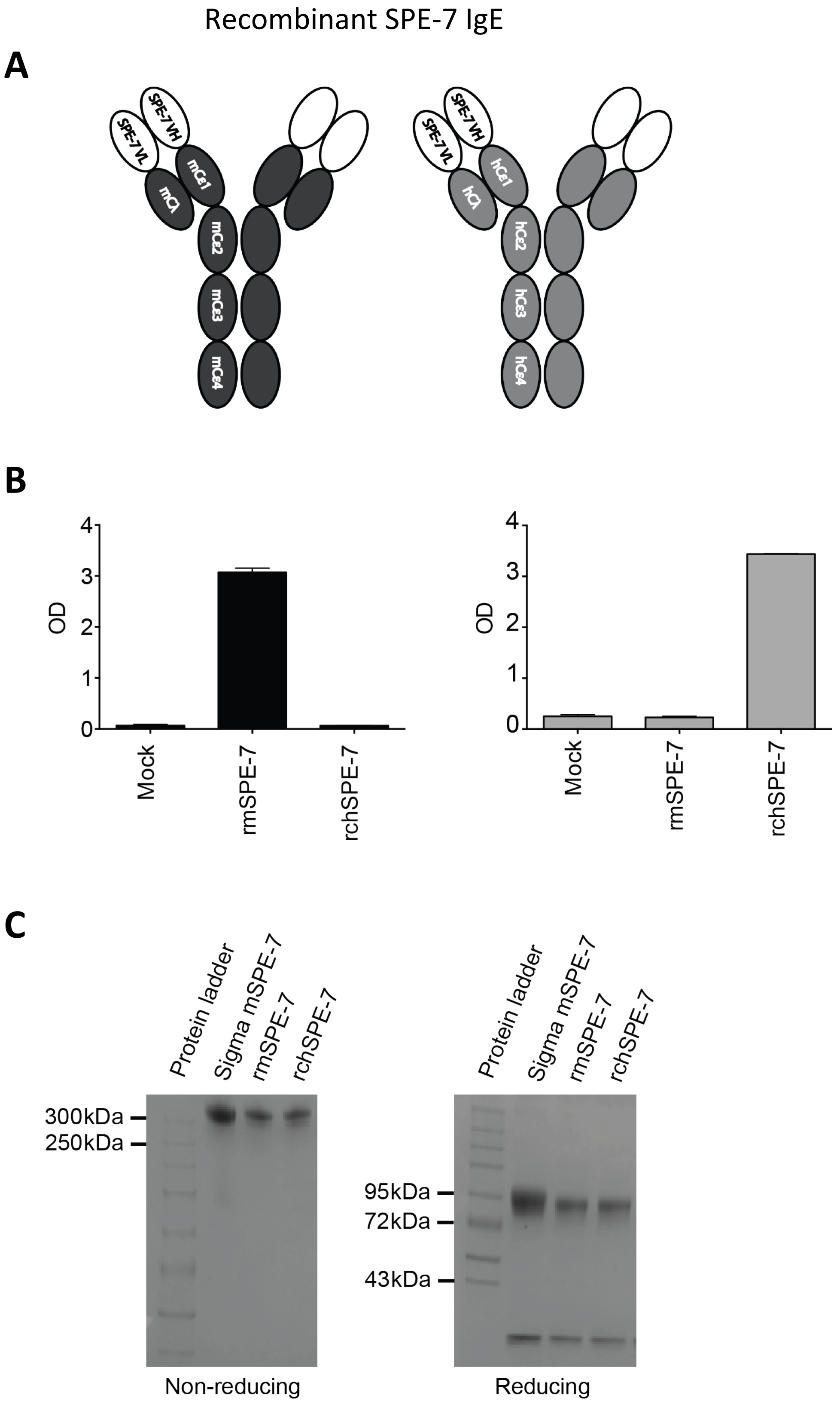

Supplementary Figure 2

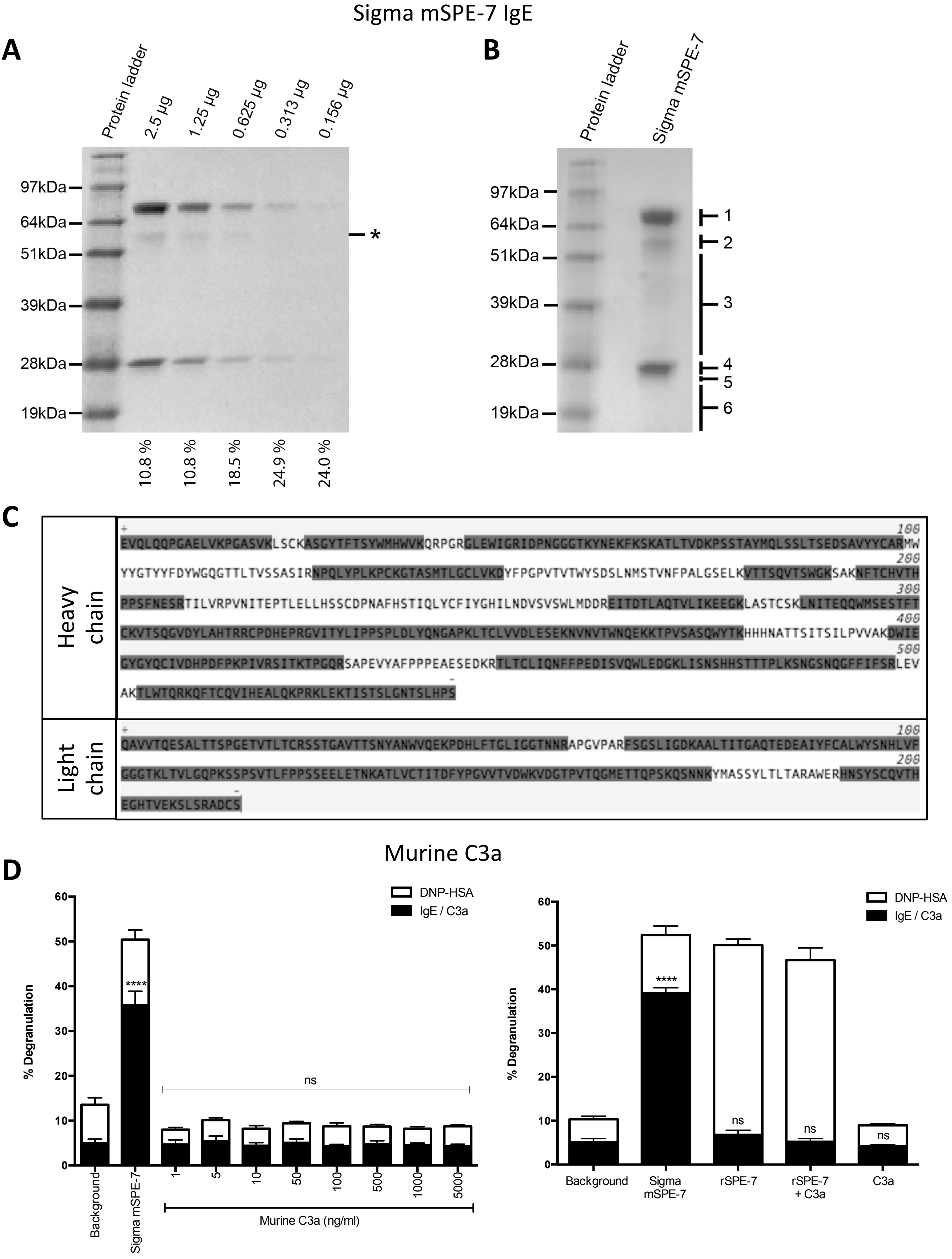

Supplementary Figure 3

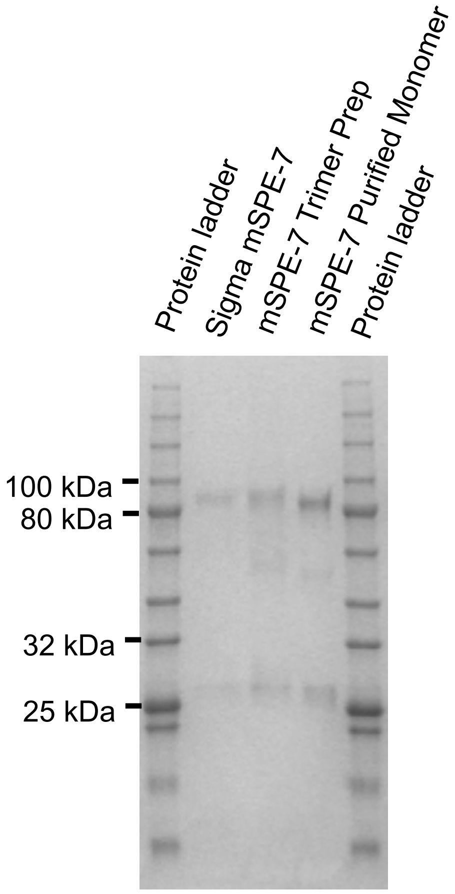

Supplementary Table 1

Excised band	No.	Identified protein	Accession no.	Molecular weight (kDa)	Unique peptide count
Band 2	1	Serum albumin *OS=Mus musculus GN=Alb PE=1 SV=3*	ALBU_MOUSE	69	30
	2	Serine protease inhibitor *A3K OS=Mus musculus GN=Serpina3k PE=1 SV=2*	SPA3K_MOUSE	47	23
	3	Ig epsilon chain C region *OS=Mus musculus PE=2 SV=2*	IGHE_MOUSE	47	19
	4	Antithrombin-III OS=Mus musculus *GN=Serpinc1 PE=1 SV=1*	ANT3_MOUSE	52	16
	5	Complement C4-B *OS=Mus musculus GN=C4b PE=1 SV=3*	CO4B_MOUSE	193	10
	6	Alpha-1-antitrypsin 1-2 *OS=Mus musculus GN=Serpina1b PE=1 SV=2*	A1AT2_MOUSE	46	8
	7	Inter alpha-trypsin inhibitor, heavy chain 4 *OS=Mus musculus GN=Itih4 PE=1 SV=2*	ITIH4_MOUSE	105	7
	8	Ig lambda-1 chain C region *OS=Mus musculus PE=1 SV=1*	LAC1_MOUSE	12	5
	9	Complement C3 *OS=Mus musculus GN=C3 PE=1 SV=3*	CO3_MOUSE	186	5
	10	Ig heavy chain V region B1-8/186-2 *OS=Mus musculus GN=Gm16709 PE=1 SV=1*	HVM07_MOUSE (+1)	15	5
Band 3	1	Serum albumin *OS=Mus musculus GN=Alb PE=1 SV=3*	ALBU_MOUSE	69	26
	2	Ig epsilon chain C region *OS=Mus musculus PE=2 SV=2*	IGHE_MOUSE	47	22
	3	Murinoglobulin-1 *OS=Mus musculus GN=Mug1 PE=1 SV=3*	MUG1_MOUSE	165	22
	4	Serine protease inhibitor A3K *OS=Mus musculus GN=Serpina3k PE=1 SV=2*	SPA3K_MOUSE	47	15
	5	Vitamin D-binding protein *OS=Mus musculus GN=Gc PE=1 SV=2*	VTDB_MOUSE	54	15
	6	Complement C4-B *OS=Mus musculus GN=C4b PE=1 SV=3*	CO4B_MOUSE	193	14
	7	Complement C3 *OS=Mus musculus GN=C3 PE=1 SV=3*	CO3_MOUSE	186	13
	8	Ig gamma-2A chain C region, A allele *OS=Mus musculus GN=Ighg PE=1 SV=1*	GCAA_MOUSE (+1)	36	13
	9	Antithrombin-III *OS=Mus musculus GN=Serpinc1 PE=1 SV=1*	ANT3_MOUSE	52	12
	10	Ig gamma-3 chain C region *OS=Mus musculus PE=1 SV=2*	IGHG3_MOUSE	44	12
Band 5	1	Ig epsilon chain C region *OS=Mus musculus PE=2 SV=2*	IGHE_MOUSE	47	15
	2	Ig lambda-1 chain C region *OS=Mus musculus PE=1 SV=1*	LAC1_MOUSE	12	9
	3	Ig kappa chain C region *OS=Mus musculus PE=1 SV=1*	IGKC_MOUSE	12	7
	4	Serum albumin *OS=Mus musculus GN=Alb PE=1 SV=3*	ALBU_MOUSE	69	6
	5	Ig gamma-2A chain C region, A allele *OS=Mus musculus GN=Ighg PE=1 SV=1*	GCAA_MOUSE (+1)	36	5
	6	Ig gamma-3 chain C region *OS=Mus musculus PE=1 SV=2*	IGHG3_MOUSE	44	5
	7	Complement C3 *OS=Mus musculus GN=C3 PE=1 SV=3*	CO3_MOUSE	186	4
	8	Ig kappa chain V-V region K2 (Fragment) *OS=Mus musculus PE=1 SV=1*	KV5A3_MOUSE	13	3
	9	Ig gamma-1 chain C region, membrane-bound form *OS=Mus musculus GN=Ighg1 PE=1 SV=2*	IGH1M_MOUSE (+1)	43	3
	10	Ig gamma-2A chain C region secreted form *OS=Mus musculus PE=1 SV=1*	GCAB_MOUSE	37	3
Band 6	1	Ig epsilon chain C region *OS=Mus musculus PE=2 SV=2*	IGHE_MOUSE	47	17
	2	Serum albumin *OS=Mus musculus GN=Alb PE=1 SV=3*	ALBU_MOUSE	69	14
	3	Desmoplakin *OS=Mus musculus GN=Dsp PE=3 SV=1*	DESP_MOUSE	333	12
	4	Ig kappa chain C region *OS=Mus musculus PE=1 SV=1*	IGKC_MOUSE	12	7
	5	Complement C4-B *OS=Mus musculus GN=C4b PE=1 SV=3*	CO4B_MOUSE	193	7
